# Silencing of brain-expressed X-linked 2 (BEX2) promotes colorectal cancer metastasis through the Hedgehog signaling pathway

**DOI:** 10.7150/ijbs.38431

**Published:** 2020-01-01

**Authors:** Yinuo Tan, Yeting Hu, Qian Xiao, Yang Tang, Haiyan Chen, Jinjie He, Liubo Chen, Kai Jiang, Zhanhuai Wang, Ying Yuan, Kefeng Ding

**Affiliations:** 1 Department of Colorectal Surgery, The Second Affiliated Hospital of Zhejiang University School of Medicine, Hangzhou, Zhejiang 310009, China.; 2 Department of Medical Oncology, The Second Affiliated Hospital of Zhejiang University School of Medicine, Hangzhou, Zhejiang 310009, China.; 3 Cancer Institute (Key Laboratory of Cancer Prevention and Intervention, China National Ministry of Education, Key Laboratory of Molecular Biology in Medical Sciences, Zhejiang Province, China), The Second Affiliated Hospital of Zhejiang University School of Medicine, Hangzhou, Zhejiang 310009, China.

**Keywords:** Colorectal cancer, BEX2, Zic2, Migration, Metastasis, Hedgehog signaling

## Abstract

The incidence of colorectal cancer is increasing, and cancer metastasis is one of the major causes of poor outcomes. BEX2 has been reported to be involved in tumor development in several types of cancer, but its role in metastatic colorectal cancer remains largely undefined. Herein, we demonstrated that BEX2 knockout resulted in enhanced migratory and metastatic potential in colorectal cancer cells both in vitro and in vivo, and re-expression of BEX2 in knockout cells could reverse the enhanced migratory capacity. RNA-Seq results indicated that the hedgehog signaling pathway was activated after BEX2 knockout; moreover, the hedgehog signaling inhibitors, GANT61 and GDC-0449 could reverse the migratory enhancement of BEX2-/- colorectal cancer cells. We also demonstrated that the nuclear translocation of Zic2 after BEX2 silencing could activate the hedgehog signaling pathway, while Zic2 knockdown abrogated the migratory enhancement of BEX2-/- cells and inhibited the hedgehog signaling pathway. In summary, our findings suggest that BEX2 negatively modulates the hedgehog signaling pathway by retaining Zic2 in the cytoplasm in colorectal cancer cells, thereby inhibiting migration and metastasis of colorectal cancer cells.

## Introduction

Colorectal cancer (CRC) is one of the most common types of cancer, with a high disease-related mortality among all cancers 1, 2. Metastasis is the leading cause of poor prognosis for CRC 3, therefore, better understanding of the mechanisms underlying metastasis suppression and identification of genes involved in metastasis suppression could definitely contribute to the treatment of CRC patients.

Brain-expressed X-linked (BEX) genes belong to a family of genes that reside on the mammalian X chromosome 4. BEX proteins have been reported to be involved in transcriptional regulation and signaling pathways in neurodegeneration, cell cycle and tumor growth 5-8. Recent reports have implicated that BEX2 is involved in tumor development and progression in several types of cancer, such as glioblastoma, glioma and breast cancer 7-9. However, BEX2 appears to exhibit diverse expression patterns and functions in different types of tumors, with conflicting evidence regarding the role of BEX2 in different cancers. BEX2 is highly expressed in glioblastomas and promotes the proliferation and survival of glioblastomas mediated by the nuclear factor-kappa B signaling 6; BEX2 can also promote cell migration and invasion in glioma 7. On the contrary, in primary glioma, BEX2 is epigenetically silenced and exhibits extensive promoter hypermethylation, and re-expression of BEX2 results in significantly suppressed tumor proliferation 10. Taken together, these data indicate that BEX2 is involved in the regulation of tumorigenesis and metastasis, and its potential functions largely depend on the cancer type and cell-specific context. In our previous studies, we demonstrated that knockdown of BEX2 significantly decreased the proliferation ability of CRC cells via the JNK/c-Jun pathway both in vitro and in vivo 11. Interestingly, our previous findings showed that in a subcutaneous xenograft model, SW620/shBEX2 cells caused higher liver metastasis rate (4/5) than SW620/Ctrl cells (1/5), suggesting that BEX2 may inhibit CRC cell metastasis. However, how BEX2 regulates CRC metastasis remains unclear.

The hedgehog signaling pathway, first identified by genetic screens in *Drosophila*, was considered to be a key regulatory pathway of cell fate determination during embryogenesis 12, 13. Aberrant activation of the hedgehog signaling pathway could enhance cell proliferation, tumor growth and metastasis in prostate, colorectal and hepatocellular carcinomas, indicating that hedgehog signaling plays a significant role in cancer development 14-18.

In this study, we investigated the role of BEX2 in CRC migration and metastasis. Our studies identified that BEX2 could inhibit Zic2 translocation into the nucleus and further suppress the hedgehog signaling pathway. BEX2 was determined as a novel regulator of the hedgehog signaling pathway that mediated migration and metastasis of CRC cells.

## Materials and Methods

### Cell culture

HEK293 cells were cultured in Dulbecco's modified Eagle's medium (DMEM) containing 10% fetal bovine serum (FBS), 100 U/mL penicillin and and 100 mg/mL streptomycin; DLD1 cells and HCT116 cells were cultured in RPMI 1640 media (Gibco, USA) supplemented with 10% FBS, 100 U/mL penicillin and 100 mg/mL streptomycin. Cells were maintained at 37°C in a humidified incubator containing 5% CO_2_. All CRC cell lines were purchased from the American Type Culture Collection (ATCC) from 2008 to 2013. All cell lines were authenticated using short tandem repeat analysis in 2015 and 2016.

### Real-time PCR and plasmid transfection

RNA was extracted from cell lines using TRIzol^@^ Reagent (Invitrogen, USA). Reverse transcription was performed using PrimeScript™ RT Master Mix (Takara, Japan), and qPCR was subsequently conducted using SYBR® Premix Ex Taq™ GC (Takara, Japan). Amplification and detection of specific products were performed with an ABI Step One Plus (PE Applied Biosystems). GAPDH was used as an internal control. The data were analyzed using the 2^-ΔΔCt^ method. All primers are listed in Supplementary Table 1.

The general protocols for plasmid construction have been previously described 19. All empty vectors were purchased from Takara Biomedical Technology (Beijing, China). All of the recombinant plasmids were identified and confirmed by DNA sequencing. Transfection of HEK293 cells was performed using Lipofectamine 2000 (Invitrogen) according to the manufacturer's protocol.

### RNA Interference

BEX2 shRNA (h) lentiviral particles (sc-60271-V) and control shRNA lentiviral particles (sc-108080) were purchased from Santa Cruz Technologies (Santa Cruz, CA, USA). Validated siRNA (sc-45881) directed against Zic2, siRNA (sc-37913) against Gli2 and control siRNA were obtained from Santa Cruz Biotechnology (CA, USA). Transfections were performed using Lipofectamine 2000 (Invitrogen) according to the modified protocol described above. The efficiency of the Zic2 siRNA was determined by western blot analysis and qPCR.

### CRISPR/Cas9

A BEX2 knockout DLD1 cell line was established by CRISPR/Cas9-mediated genome editing technology. The target sequences for CRISPR interference were designed by Shanghai Innovative Cellular Therapeutics Co., Ltd., Shanghai, China. The target sequence for human BEX2 was GTCCATTTTCTCTCTGTCTCC. The clones were screened by sequencing.

### Western immunoblotting

Cell samples were lysed in lysis buffer (Thermo Scientific, Rockford, IL, USA) containing Complete Protease Inhibitor Cocktail and Phosphatase Inhibitor Cocktail (Thermo Scientific, Rockford, IL, USA). Immunoblotting was carried out according to previously described methods. All antibodies are listed in Supplementary Table 2.

### Transwell migration and Matrigel invasion assays

The effect of BEX2 on the migratory and invasive ability of cells was determined using the Transwell polycarbonate membrane (3422, Costar, New York, NY, USA), which was originally described as the Boyden chamber assay.

### Confocal microscopy

All cells were seeded on coverslips in 12-well plates. After transfection for 24 hours, the cells were fixed, permeabilized, and incubated with the indicated antibodies. The details of the experimental procedure were described previously. Images were obtained using a Zeiss LSM 710 laser-scanning confocal imaging system (Carl Zeiss AG, Oberkochen, Germany).

### Xenografts

The orthotopic mouse model of CRC was established using cecal wall injection technique according to previous description 20, 21. In brief, after anesthesia, the cecum of nude mouse was exteriorized via laparotomy. A total of 1×10^6^ CRC cells (50 μl) were injected into the cecal wall using a 27-gauge needle. The cecum was subsequently returned to the abdominal cavity and closed with running sutures.

Another mouse model, the hemi-spleen injection model, was also used. After anesthesia and laparotomy, the spleen was first split into two parts with two titanium clips, leaving the vascular pedicle intact for each half of the spleen. BEX2^-/-^ DLD1 cells and DLD1 control cells (1×10^6^ CRC cells per mouse) were inoculated into one hemi-spleen, which was further resected. After injection for 45 days, an in vivo imaging system was used to detect liver metastases.

All mice were monitored twice daily. Overall survival was analyzed using the Kaplan-Meier method. All animal experiments were approved by the Animal Care and Use Committee of Zhejiang University.

### RNA sequencing and analysis

Total RNA was extracted using TRIzol, and an Illumina TruSeq RNA Sample Prep Kit (Cat#FC-122-1001) was used with 1 µg of total RNA for the construction of sequencing libraries. RNA libraries were prepared for sequencing using standard Illumina protocols, and RNA sequencing was subsequently performed using the Illumina HiSeq 3000 platform. Finally, differentially expressed genes were screened using DESeq (version 1.22.1).

### Statistical analysis

Analysis was performed with SPSS 22.0 software for Windows. Comparisons between two groups were carried out using a t-test for independent samples. A *p* value less than 0.05 was considered as statistical significance.

## Results

### Knockout of BEX2 in colorectal cancer cells using CRISPR/Cas9

**Figure 1:**
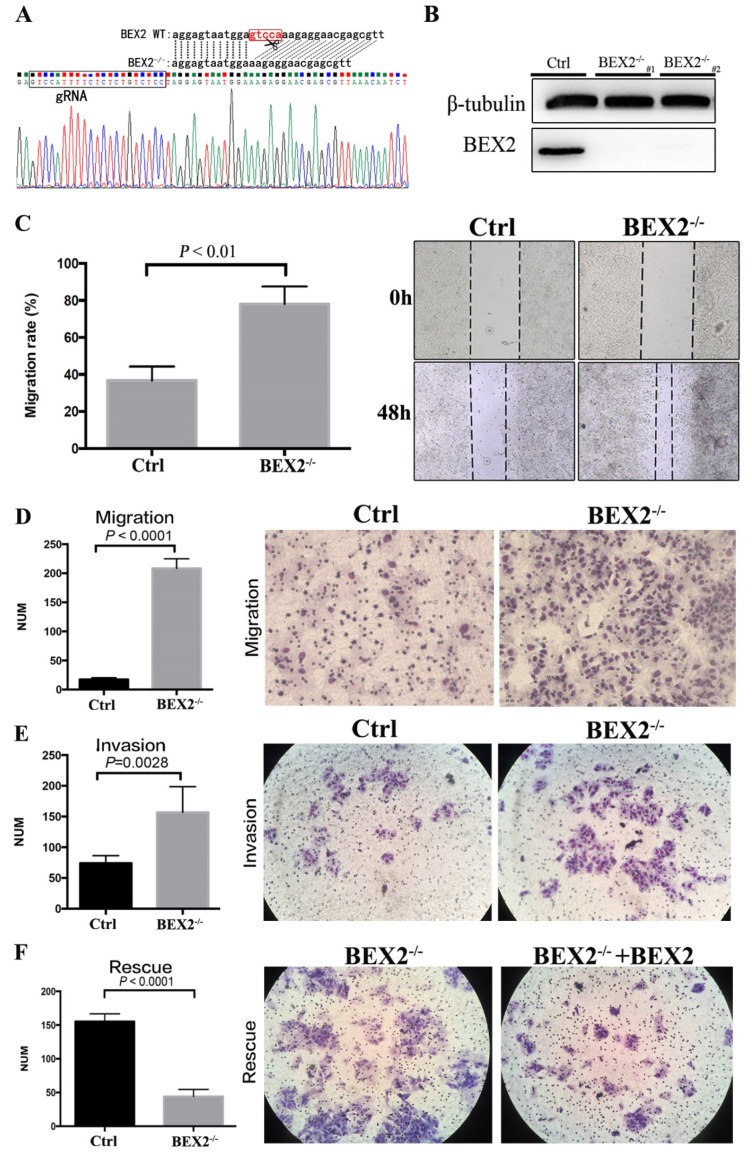
** BEX2 knockout via CRISPR/Cas9 in CRC cells enhanced mobility, migration and invasion. (A)** Sequence results revealed that 5 bps in the BEX2 gene were completely deleted in BEX2^-/-^ DLD1 cells. **(B)** Protein expression of BEX2 in BEX2^-/-^ DLD1 cells and control cells. **(C)** Wound-healing assay. (left panel, average counts of results of triplicate; right panel, representative pictures) (magnification, ×200). **(D)** Cell migration assays using Transwell membranes. (left panel, average counts from five random microscopic fields; right panel, representative images of invasion chambers) (magnification, ×200). **(E)** Cell invasion assays using Matrigel-pre-coated Transwell membranes (left panel, average counts from five random microscopic fields; right panel, representative images of invasion chambers) and BEX2^-/-^ DLD1 cells and control cells. (magnification, ×200). **(F)** Cell migration assays using Transwell membranes (left panel, average counts from five random microscopic fields; right panel, representative images of invasion chambers) and BEX2 re-expression in BEX2 knockout cells, BEX2^-/-^ DLD1+BEX2 cells and BEX2^-/-^ DLD1 cells. (magnification, ×200).

The CRISPR/Cas9 system was employed to stably knock out BEX2 in CRC cell line DLD1, in consideration of the relatively high protein expression of BEX2 in DLD1 cell line compared to other CRC cell lines. The sequence results (Figure 1A) showed that 5 bps in the translation initiation region of BEX2 gene were completely deleted in BEX2^-/-^ DLD1 cells, therefore, the whole amino acid sequence of BEX2 could not be translated because of the frameshift mutation. In addition, the protein expression level of BEX2 and knockout efficiency of BEX2^-/-^ DLD1 cells were confirmed by western blotting (Figure 1B).

### BEX2 inhibited the mobility, migration and invasion of colorectal cancer cells

In a preliminary experiment, SW620 cells were transfected with lentivirus shRNA and control vector. As a result, BEX2 expression was reduced by 70% in SW620/shBEX2 cells compared with that in control cells (SW620/Ctrl cells) according to western blotting and qPCR, as described previously. Interestingly, in our subcutaneous models, higher liver metastasis rate (4/5) was observed in mice inoculated with SW620/shBEX2 cells than those inoculated with SW620/Ctrl cells (1/5) (Supplementary Table 2). And the liver metastasis lesion was confirmed by HE staining (Supplementary Figure 1).

Intriguingly, the above outcomes indicated that knockdown of BEX2 was more likely to cause liver metastases of CRC. Thus, we aimed to further confirm whether BEX2 played a causal role in regulating CRC cell mobility, migration and invasion ability. To this end, the effects of BEX2 on the mobility, migration and invasion abilities of CRC cells were first examined in vitro.

The migration of BEX2^-/-^ DLD1 cells was significantly enhanced compared with that of control cells in the wound-healing assay (Figure 1C). Consistently, the Transwell migration assay demonstrated that BEX2^-/-^ DLD1 cells had enhanced migration ability than control cells (Figure 1D). Similar results were also observed in the Transwell invasion assay (Figure 1E). To confirm the inhibitory effect of BEX2 on migration and invasion, another colon cancer cell line, HCT116, was used. Consequently, HCT116/shBEX2 cells displayed significantly enhanced migration and invasion ability compared to HCT116/ctrl cells (Supplementary Figure 2A and B).

The above results showed that BEX2 silencing could lead to enhanced migration and invasion capacity of CRC cells. To further validate the above outcomes, BEX2 was re-expressed in BEX2^-/-^ DLD1 cells by transfecting with BEX2-overexpression lentivirus, which was thereafter named as BEX2^-/-^ DLD1+BEX2 cells. The BEX2^-/-^ DLD1+BEX2 cells showed suppressed migration ability than BEX2^-/-^ DLD1 cells (Figure 1F), further indicating that BEX2 inhibited the migration ability of CRC cells. Altogether, these results suggested that BEX2 can inhibit the mobility, migration and invasion of CRC cells in vitro.

### BEX2 silencing promoted colorectal cancer cell liver metastasis in vivo

**Figure 2:**
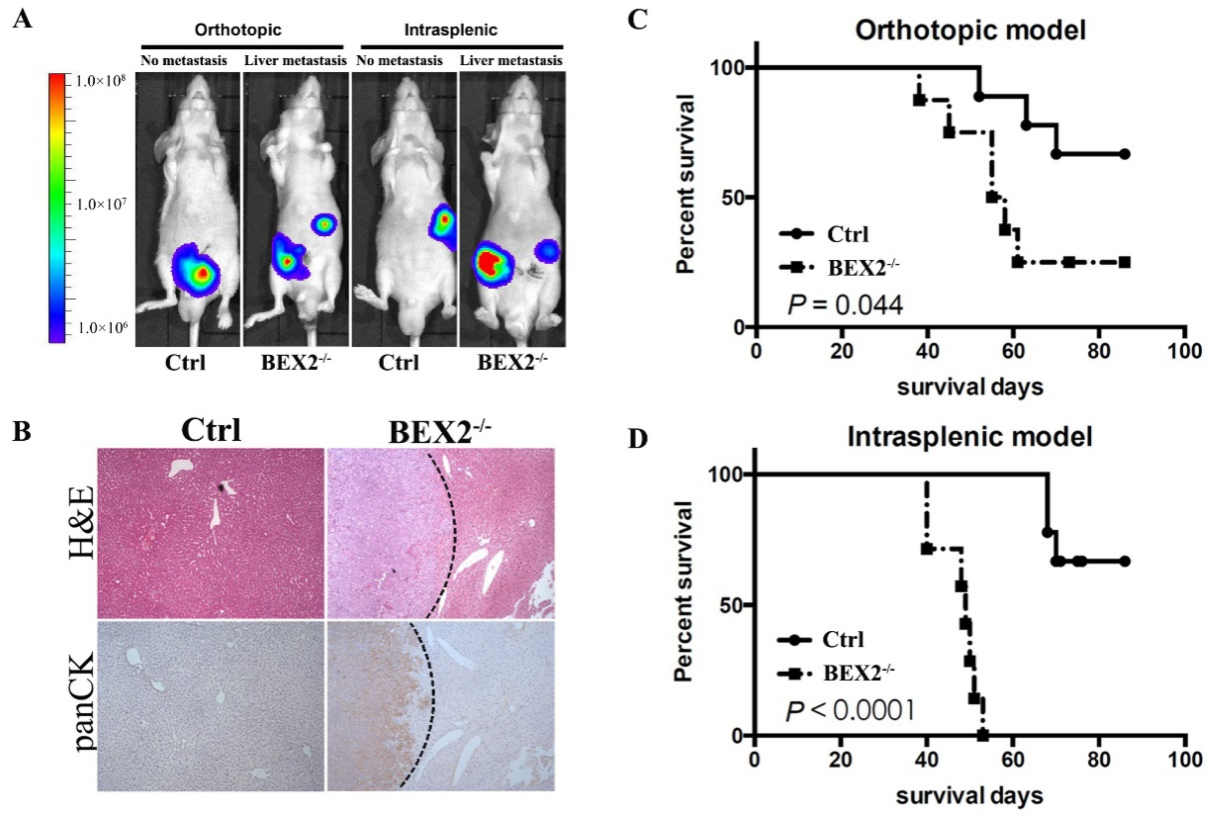
** BEX2 knockout promoted CRC metastasis in vivo. (A)** In vivo bioluminescent images showed liver metastasis in mice with BEX2^-/-^ DLD1 cells and control cells in both orthotopic and intrasplenic models. **(B)** Hematoxylin and eosin (H&E) staining and pan-cytokeratin (panCK) staining in normal liver tissue and liver metastatic tumors. (magnification, ×50). **(C)** Mouse survival curves for each group of the orthotopic model. (log-rank test) **(D)** Mouse survival curves for each group of the intrasplenic model. (log-rank test).

Afterwards, we assessed the effects of BEX2 on tumor metastasis in vivo by using mouse models. Mice injected with BEX2^-/-^ DLD1 cells displayed higher more liver metastasis rate than the mice in the control group (Figure 2A and B).

In the hemi-spleen injection model, we found that 100% of the BEX2^-/-^ DLD1 mice had metastases, whereas only 60% of the control mice had metastases in their livers (Table 1). Another mouse model 20, 21, the orthotopic model, was also used to determine the effects of BEX2 on liver metastasis. As shown in Table 1 and Figure 2A, more metastatic lesions were detected in BEX2^-/-^ DLD1 mice than the control mice. All metastatic lesions were confirmed by immunohistochemistry assay stained with pan-cytokeratin antibody (Figure 2B).

The survival outcomes of BEX2^-/-^ DLD1 mice and control mice were also recorded and evaluated, which demonstrated that worse survival of BEX2^-/-^ DLD1 mice than that of control mice (Figure 2C and D).

Taken together, these results demonstrated that BEX2 silencing exerted a potent impact on poor survival by inducing CRC metastasis.

### BEX2 silencing activated the hedgehog signaling cascade

**Figure 3:**
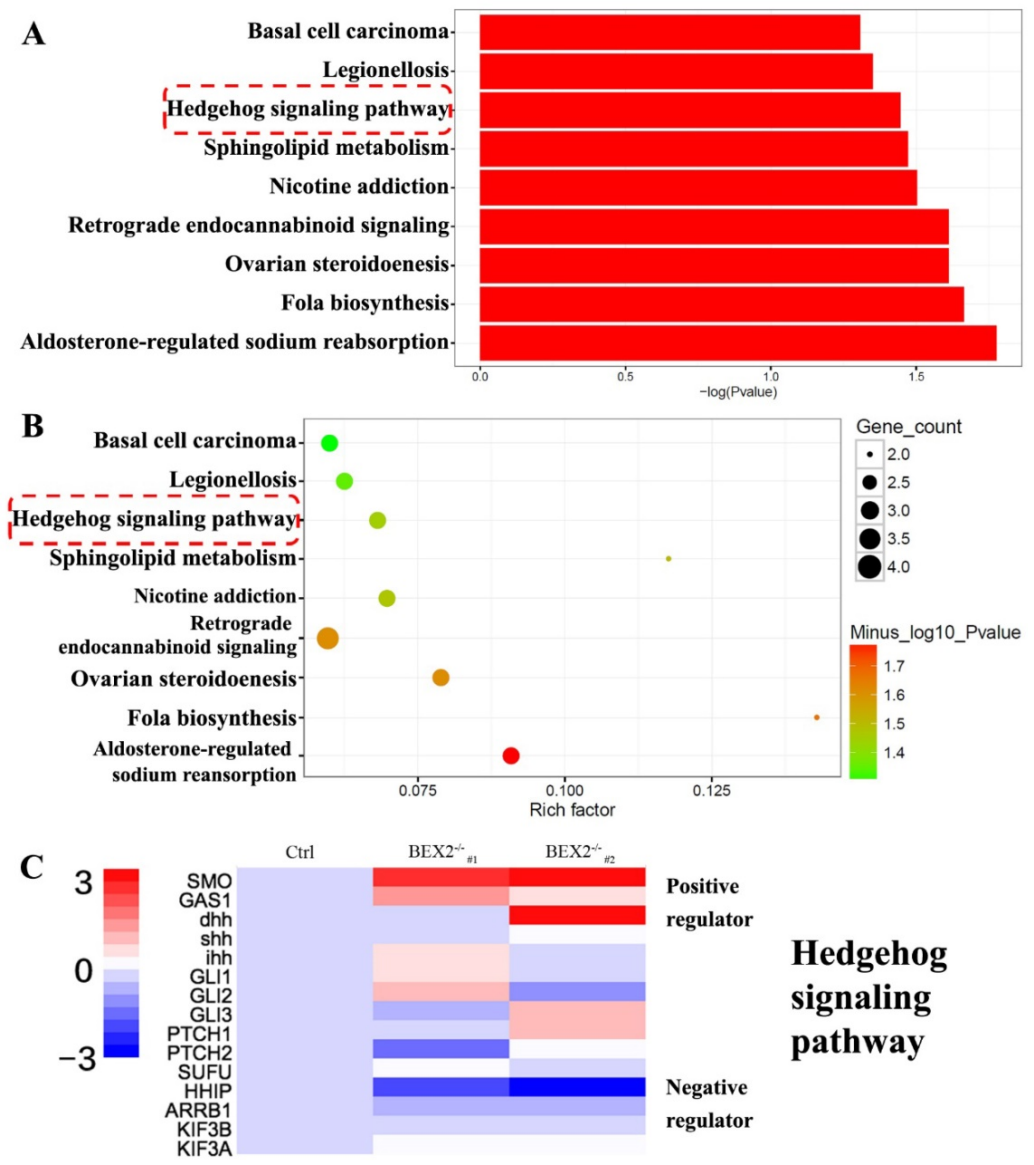
** RNA-Seq results after BEX2 was knocked out. (A)** Histogram of significantly enriched KEGG pathways. **(B)** Scatter diagram of significantly enriched KEGG pathways. **(C)** Some important gene expression changes in the Hedgehog signaling cascade.

To better understand the mechanism of increased liver metastases in the BEX2^-/-^ group, RNA-Seq technology was used to analyze the gene expression profiles of BEX2^-/-^ DLD1 cells and DLD1 control cells. RNA-Seq analysis (data were uploaded to the GEO database, GSE112590) revealed a variety of genes with altered expression. GO term analysis and KEGG pathway analysis were subsequently performed, showing that several pathways, especially the hedgehog signaling pathway, played an important role (Figure 3A-C).

**Figure 4:**
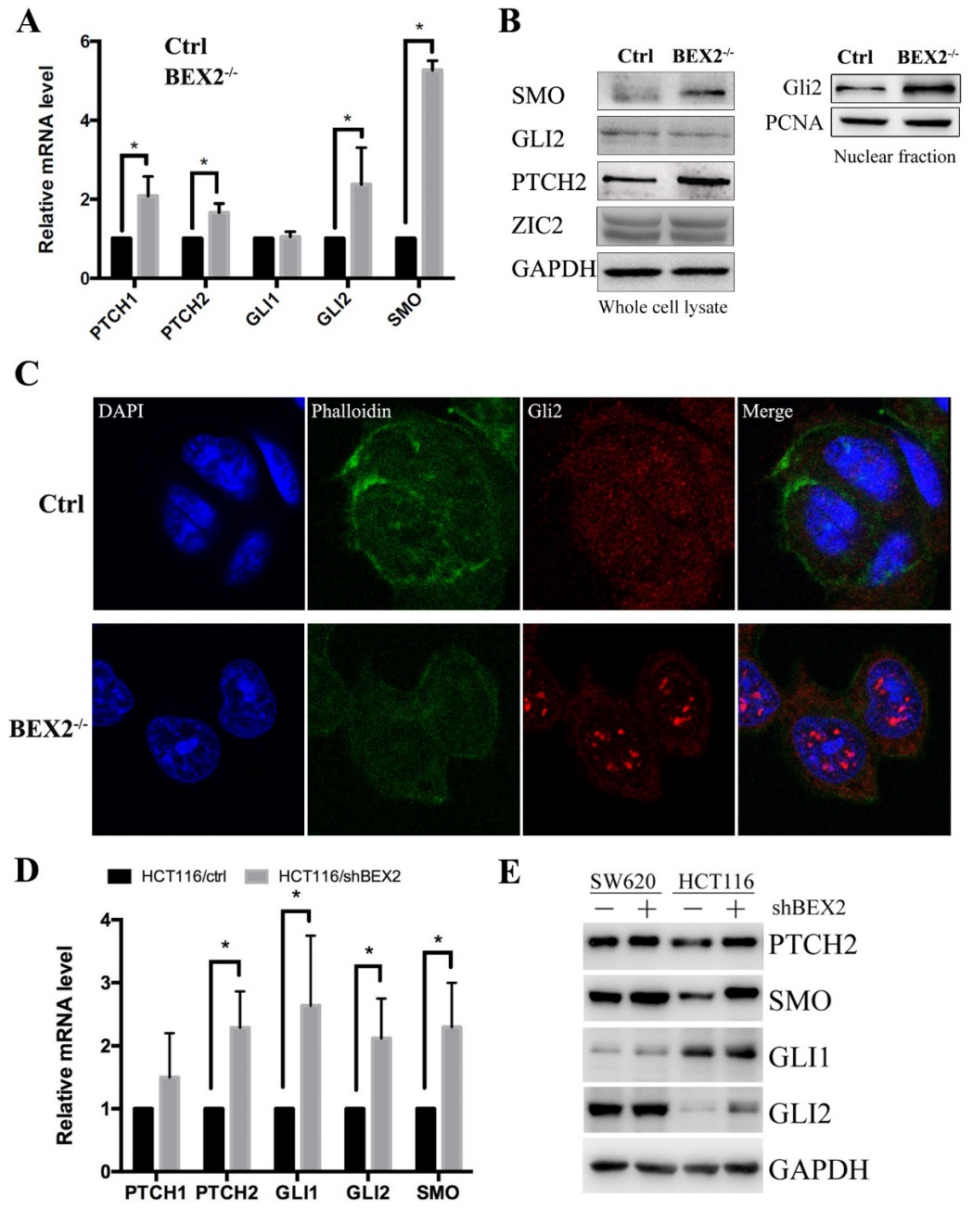
** Validation of Hedgehog signaling activation after BEX2 silencing. (A)** The mRNA levels of PTCH1, PTCH2, GLI2 and SMO were significantly increased after BEX2 knockout. * P < 0.05. **(B)** Western blotting of SMO, GLI2, PTCH2 and ZIC2 in both BEX2^-/-^ DLD1 cells and control cells. **(C)** Immunofluorescence assay of GLI2 in BEX2^-/-^ DLD1 cells and control cells. Nuclei were stained with DAPI (blue), and the cell skeleton was stained with phalloidin (green). Representative images were shown (magnification, ×680). **(D)** The mRNA levels of PTCH2, GLI1, GLI2 and SMO were significantly increased after BEX2 knockdown in HCT116 cells. * P < 0.05. **(E)** Western blotting results showed that the protein expression of PTCH2, SMO, GLI1, and GLI2 was higher in SW620/shBEX2 and HCT116/shBEX2 cells than that in control cells.

To confirm the RNA-Seq results, qPCR was used to validate several important components of the hedgehog signaling pathway, including SMO, PTCH1, PTCH2, Gli1, and Gli2 (Figure 4A). In the hedgehog signaling cascade, SMO could trigger target gene transcription through the Gli family of transcription factors 22. Western blot showed that SMO expression was increased in BEX2^-/-^ DLD1 cells (Figure 4B). Two important target genes, Ptch1 and Ptch2, were both found to be increased at the mRNA level (Figure 4A). Moreover, the protein expression of Ptch2 was also increased in BEX2^-/-^ DLD1 cells (Figure 4B). Then, we explored Gli family protein levels in BEX2^-/-^ DLD1 cells and control cells. As a result, Gli1 was hardly detected, while Gli2 expression was not significantly different in the whole cell lysates between BEX2^-/-^ DLD1 cells and control cells (Figure 4B). In view that Gli2 is a transcription factor that may play a role mainly in the nucleus, we employed a fractional assay to separate the nuclear fraction and cytoplasm fraction. As shown in Figure 4B, Western blot assay revealed that Gli2 expression was increased in the nuclear fraction of BEX2^-/-^ DLD1 cells (Figure 4B). In addition, immunofluorescence assay was utilized to confirm our finding, which showed enhanced translocation of Gli2 into the nucleus in BEX2^-/-^ DLD1 cells (Figure 4C). These results all indicated that the hedgehog signaling pathway was activated in BEX2^-/-^ DLD1 cells compared with that in DLD1 control cells.

To confirm the relationship between BEX2 and the hedgehog signaling pathway, qPCR and Western blotting were performed to compare several important components of the hedgehog signaling pathway in BEX2 knockdown cells and control cells. The results showed that the mRNA levels of several important genes in the hedgehog signaling pathway, including PTCH2, Gli1, Gli2, and SMO, were significantly higher in HCT116/shBEX2 cells than those in HCT116/ctrl cells (Figure 4D). With respect to their protein levels, Western blot results showed that PTCH2, Gli1, Gli2 and SMO expression levels were lower in BEX2 knockdown SW620 and HCT116 cells than in control cells (Figure 4E). Additionally, we examined the relationship between BEX2 and Gli1 in the GSE3629 cohort from the GEO database, showing that BEX2 expression was negatively correlated with Gli1 expression (R value=-0.642, p value=2.1e-15, Supplementary Figure 3).

### Hedgehog signaling inhibitors suppressed migration in BEX2 knockout colorectal cancer cells

**Figure 5:**
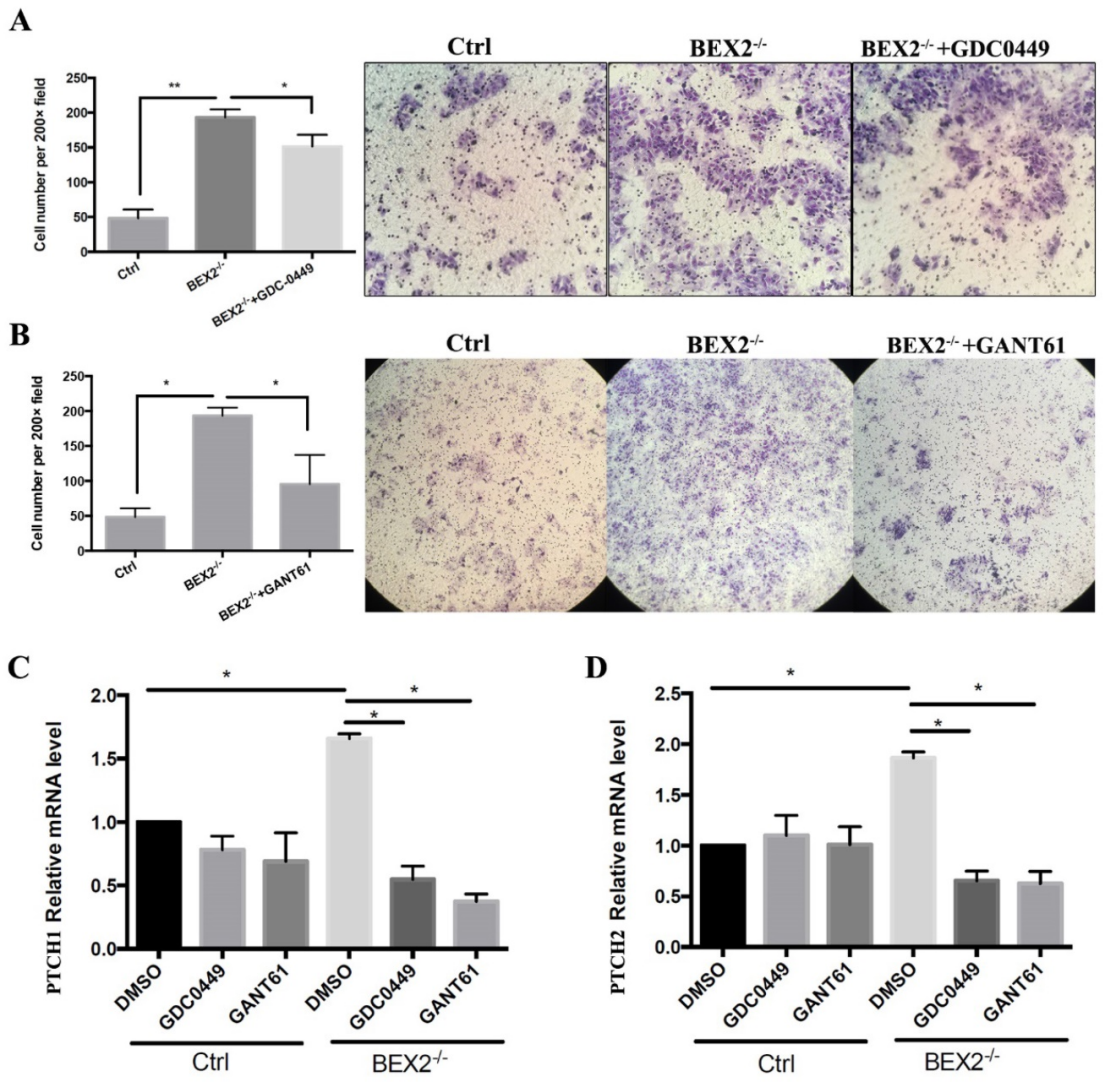
** Hedgehog signaling inhibitors abrogated the enhanced migration ability in BEX2^-/-^ DLD1 cells. (A)** Cell migration assays using Transwell membranes after GDC-0449 treatment. * P < 0.05. (left panel, average counts from five random microscopic fields; right panel, representative images of invasion chambers) (magnification, ×200). **(B)** Cell migration assays using Transwell membranes after GANT61 treatment. * P < 0.05. (left panel, average counts from five random microscopic fields; right panel, representative images of invasion chambers) (magnification, ×100). **(C)** Relative mRNA level of PTCH1 in BEX2^-/-^ DLD1 cells and control cells treated with DMSO, GDC-0449 or GANT61. * P < 0.05. **(D)** Relative mRNA level of PTCH2 in BEX2^-/-^ DLD1 cells and control cells treated with DMSO, GDC-0449 or GANT61. * P < 0.05.

The hedgehog signaling pathway was activated in CRC cells after BEX2 silencing; thus, we asked whether hedgehog signaling inhibitors could abrogate the enhanced migration ability in BEX2^-/-^ DLD1 cells. Thus, two types of hedgehog signaling inhibitors, GANT61 (Gli protein inhibitor) and GDC-0449 (SMO inhibitor) were used, demonstrating that the upregulation of migration ability caused by BEX2 silencing could be blocked by GANT61 and partially blocked by GDC-0449 (Figure 5A and B).

Next, we asked whether the expression of target genes of the hedgehog signaling pathway, PTCH1 and PTCH2, could be rescued by these inhibitors. qPCR showed that the increased mRNA expression of PTCH1 and PTCH2 in BEX2^-/-^ DLD1 cells could be rescued and downregulated after treatment with GANT61 and GDC-0449, while the mRNA expression of PTCH1 and PTCH2 mRNA was not significantly altered in DLD1 control cells after treatment with GANT61 and GDC-0449 (Figure 5C and D). These results suggested that the enhanced hedgehog signaling in BEX2 knockout cells could be rescued by hedgehog signaling inhibitors.

Gli family proteins are key transcriptional factors that could represent hedgehog signaling activity 12. Gli2 was found to be increased in the nuclear fraction of BEX2^-/-^ DLD1 cells. We further applied siRNA targeting Gli2 to knock down Gli2 in BEX2^-/-^ DLD1 cells, followed by Western blotting to confirm the knockdown efficiency (Supplementary Figure 4A). Moreover, Transwell migration assays and invasion assays both showed that knockdown of Gli2 could inhibit the migration ability of BEX2^-/-^ DLD1 cells (Supplementary Figure 4B). We hypothesized that BEX2 may interact with Gli2, and co-immunoprecipitation assay was performed. However, the result was negative (data not shown), indicating that Gli2 may not directly interact with BEX2.

### BEX2 silencing enhanced hedgehog signaling by promoting Zic2 translocation into the nucleus

As Gli2 and BEX2 may not interact directly, we sought other possible regulatory mechanism. The Zic family and Gli family have been found to cooperate with each other and control the gene transcriptional activity and subcellular localization of each other 23. Zic family members, including Zic1, Zic2 and Zic3, share five highly conserved C2H2 zinc finger (ZF) motifs 24. And these domains show remarkably homologous with those of Gli family proteins 25.

**Figure 6:**
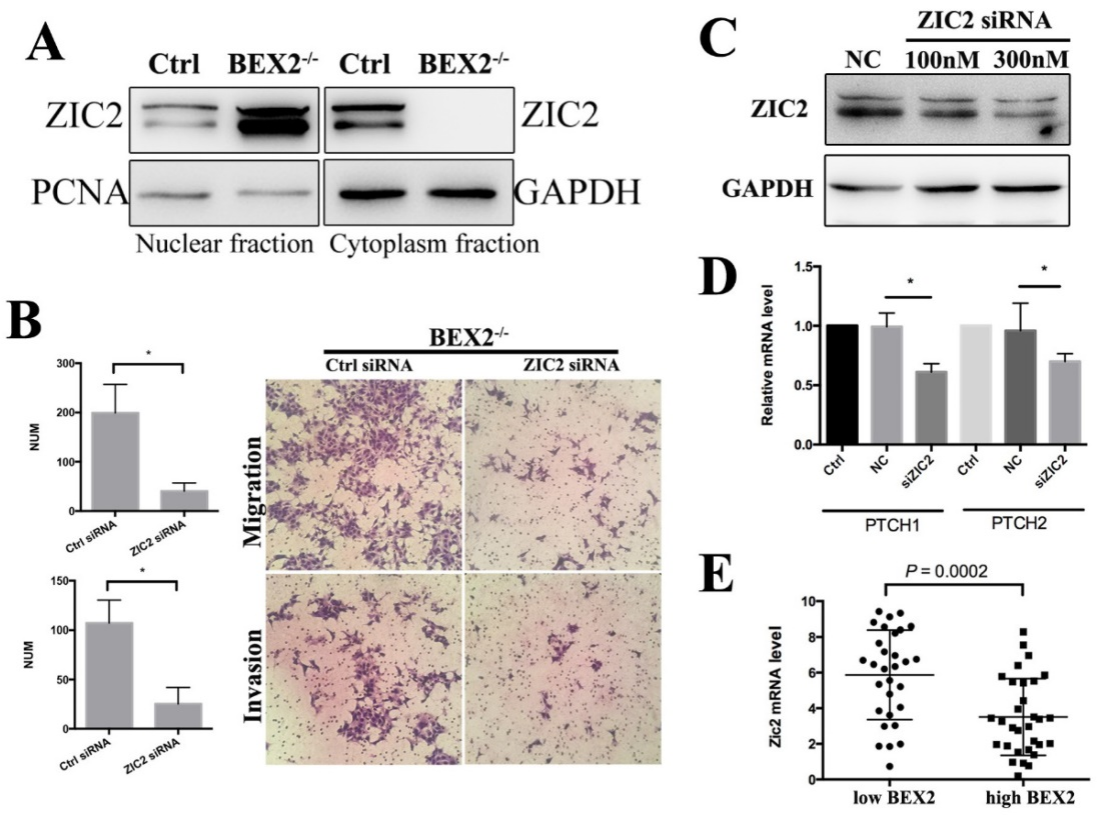
** Zic2 knockdown abrogated the enhanced migration ability in BEX2^-/-^ DLD1 cells. (A)** Fractional assay of ZIC2 in nuclear and cytoplasm fractions of BEX2^-/-^ DLD1 cells and control cells. **(B)** Cell migration and invasion assays using Transwell membranes after control siRNA or ZIC2 siRNA treatment. **(C)** Western blotting for ZIC2 after treatment with ZIC2 siRNA or normal control (NC) siRNA. **(D)** The mRNA expression of PTCH1 and PTCH2 was downregulated after ZIC2 siRNA treatment in BEX2^-/-^ DLD1 cells. * P < 0.05. (left panel, average counts from five random microscopic fields; right panel, representative images of invasion chambers) (magnification, ×200). **(E)** The mRNA level of ZIC2 in tumors with low BEX2 expression and high BEX2 expression.

Zic2 is well known to play an important role in the hedgehog signaling pathway as a transcriptional co-activator. In whole cell lysates, there was no significant difference of Zic2 between BEX2^-/-^ DLD1 cells and control cells (Figure 4B). The fractional assay showed that Zic2 was distributed in both the cytoplasm and nuclei in DLD1 control cells; however, after BEX2 silencing, Zic2 appeared almost exclusively in the nuclei of BEX2^-/-^ DLD1 cells (Figure 6A), showing the translocation of Zic2 translocated to the nucleus after BEX2 silencing. Thus, we assessed whether Zic2 played a crucial role in activating the hedgehog signaling pathway in BEX2^-/-^ DLD1 cells. siRNA targeting Zic2 was used to knock down Zic2 in BEX2^-/-^ DLD1 cells, followed by Western blotting and qPCR to confirm the knockdown efficiency (Figure 6C). Transwell migration assays and invasion assays both showed that knockdown of Zic2 could inhibit the migration ability of BEX2^-/-^ DLD1 cells (Figure 6B). In addition, the expression of hedgehog target genes (PTCH1 and PTCH2) was also downregulated after Zic2 was knocked down in BEX^-/-^ cells (Figure 6D). These results collectively suggested that Zic2 may be crucially involved in enhancing hedgehog signaling activity after BEX2 silencing.

## Discussion

In our study, we demonstrated that BEX2-knockout or -knockdown cells showed enhanced migration and invasion abilities in vitro and enhanced metastasis ability in vivo. With the re-expression of BEX2 in BEX2^-/-^ DLD1 cells, migration ability was re-suppressed. These results indicated that BEX2 can inhibit CRC cell migration. RNA-Seq data showed that BEX2 knockout mainly affected the hedgehog signaling pathway. After treatment with hedgehog signaling inhibitors (GANT61 and GDC-0449), the enhanced migration ability of BEX2^-/-^ DLD1 cells was abrogated. We also found that the key molecule Zic2 played an important role in activating the hedgehog signaling pathway after BEX2 silencing. Zic2 has been reported to be a modulator of the hedgehog signaling pathway and can physically and functionally interact with Gli proteins through their zinc finger domains. In our study, it was also demonstrated that after BEX2 was silenced, Zic2 was translocated to the nucleus. After Zic2 was knocked down, the enhanced migration and invasion abilities could be rescued, and the hedgehog signaling pathway could be inhibited.

Cancer cell metastasis involves multiple biological steps, and cancer cell migration and invasion are necessary for the malignant progression of cancer. Er Nie et al 26 reported that BEX2 downregulation decreased the migration and invasion in glioma cells by decreasing the nuclear and cytoplasmic protein levels of β-catenin. In our study, the complete depletion of BEX2 led to enhanced migration and invasion capacity of CRC cells. The mRNA and protein levels of β-catenin in DLD1 and BEX2^-/-^ DLD1 cells were not obviously different, regardless of their nuclear or cytoplasmic location (data not shown). Wnt/β-catenin signaling has been reported to play an important role in promoting cancer metastasis; however, our study did not show Wnt/β-catenin signaling activation after BEX2 depletion in CRC cells.

Accumulating evidence has indicated the important role of hedgehog signaling pathway dysregulation in metastasis 27, 28. Components of the hedgehog signaling pathway, such as SMO, Gli1, and Gli2, are deregulated in various cancers, and their expression levels are correlate with tumor progression and metastasis 14, 22, 29, 30. Aberrant activation of the hedgehog signaling pathway may promote colon cancer growth, recurrence, metastasis and stem cell survival and expansion 31, 32. Our study showed that when BEX2 was knocked out, the hedgehog signaling pathway could be activated. However, how the hedgehog signaling pathway is activated after BEX2 silencing in CRC is unknown. It is well known that Gli family members are key transcriptional factors in the hedgehog signaling pathway 13. We demonstrated that the expression of Gli2 was higher in the nuclei of BEX2^-/-^ DLD1 cells than that in the nuclei of DLD1 cells, while the protein expression of Gli1 was barely detectable (data not shown). We assumed that BEX2 could interact with Gli2 to activate the hedgehog signaling pathway, but the co-immunoprecipitation results for BEX2 and Gli2 (data not shown) were negative; therefore, BEX2 and Gli2 did not have a direct interaction. The Gli family and the Zic family have been found to cooperate with each other and control the gene transcriptional activity and subcellular localization of each other 23, 33. Zic2 has been reported to enhance hedgehog signaling activity through the interaction with and retention of Gli1 in the nucleus 34; while in our study, Gli1 protein level was rarely detectable, indicating that Gli1 may not be the key transcriptional factor in our CRC cells. Our results demonstrated that Zic2 knockdown could attenuate the enhanced migration and invasion of BEX2^-/-^ DLD1 cells and inhibit hedgehog signaling. In BEX2^-/-^ DLD1 cells, Zic2 was translocated into the nuclei. Therefore, we concluded that BEX2 silencing can promote the activation of hedgehog signaling pathway and Zic2 may regulate this process.

In our previous study, we analyzed the expression level of BEX2 in 290 CRC patients in the GSE14333 sample cohort and observed that BEX2 expression levels were significantly higher in Dukes D stage patients than in other stage patients 11. This result conflicts with the metastasis-inhibiting role of BEX2 in our current study. In fact, cancer metastasis is a complicated process driven by multiple genes and multiple steps. A single gene, BEX2 may not have a determinant role such that it can completely inhibit metastasis, while BEX2 may have certain regulatory effect on tumor masses in patients with Dukes D stage, especially in relation to hedgehog signaling molecules. We analyzed the expression of Zic2 in patients with Dukes D stage from the GSE14333 sample cohort, revealing that the BEX2 low expression group had significantly higher Zic2 expression than the BEX2 high expression group (Figure 6E). This finding indicated that BEX2 may have some relationship with Zic2, and more studies are needed to investigate the specific subgroup of tumor patients with Dukes D stage can be influenced by BEX2. We additionally explored the location of Zic2 after restoring BEX2 levels in BEX^-/-^ cells; however, Zic2 was not found to return to the cytoplasm after restoring BEX2 in BEX^-/-^ cells. There may be some underlying regulatory mechanism between Zic2 and BEX2, which deserves further investigation.

In this study, we identified BEX2 as a novel regulator of the hedgehog signaling pathway in the colorectal metastasis process. BEX2 could suppress hedgehog signaling activity and CRC cell metastasis. The hedgehog signaling pathway has been reported to be aberrantly activated in some CRC patients, and BEX2 may be a candidate target for inhibiting hedgehog signaling. Further studies will be necessary to investigate the potential of BEX2 as an effective therapeutic target in metastatic CRC, especially patients with aberrantly activated hedgehog signaling.

In conclusion, we have demonstrated that BEX2 is involved in inhibiting CRC metastasis and BEX2 silencing is correlated with hedgehog signaling activity. Notably, we have shown Zic2 involvement in the molecular mechanism of enhanced hedgehog signaling activity in BEX2-silenced CRC cells.

## Supplementary Material

Supplementary figures and tables.

## Figures and Tables

**Table 1:** Quantitative analysis of liver metastasis in BEX2^-/-^ DLD1 mice and control mice

		Mice with liver metastases	Mice without liver metastases
Splenic model	Ctrl	6	4
	BEX2-/-	10	0
Orthotopic model	Ctrl	5	5
	BEX2-/-	8	1
