# Supplementary material for: Silencing of brain-expressed X-linked 2 (BEX2) promotes colorectal cancer metastasis through the Hedgehog signaling pathway
Source: Int J Biol Sci. 2020 Jan 1;16(2):228–38. doi: 10.7150/ijbs.38431 (PMC6949152; doi:10.7150/ijbs.38431)
Supplement: Supplementary file 1 — Supplementary figures and tables. [file ijbsv16p0228s1.pdf]

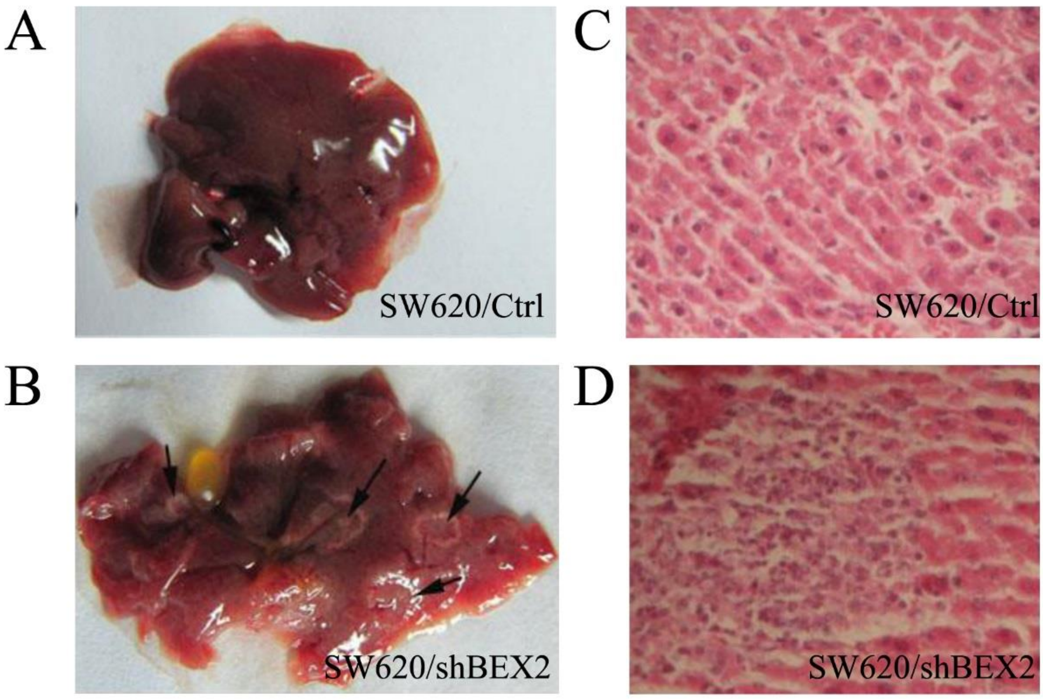

Supplementary Figure 1

A and B, Representative images showing liver metastases in control and shBEX2 mice. (arrows indicate liver metastasis)

C and D, Representative images of hematoxylin and eosin (H&E) staining showing liver metastases in control and shBEX2 mice. (magnification, ×200)

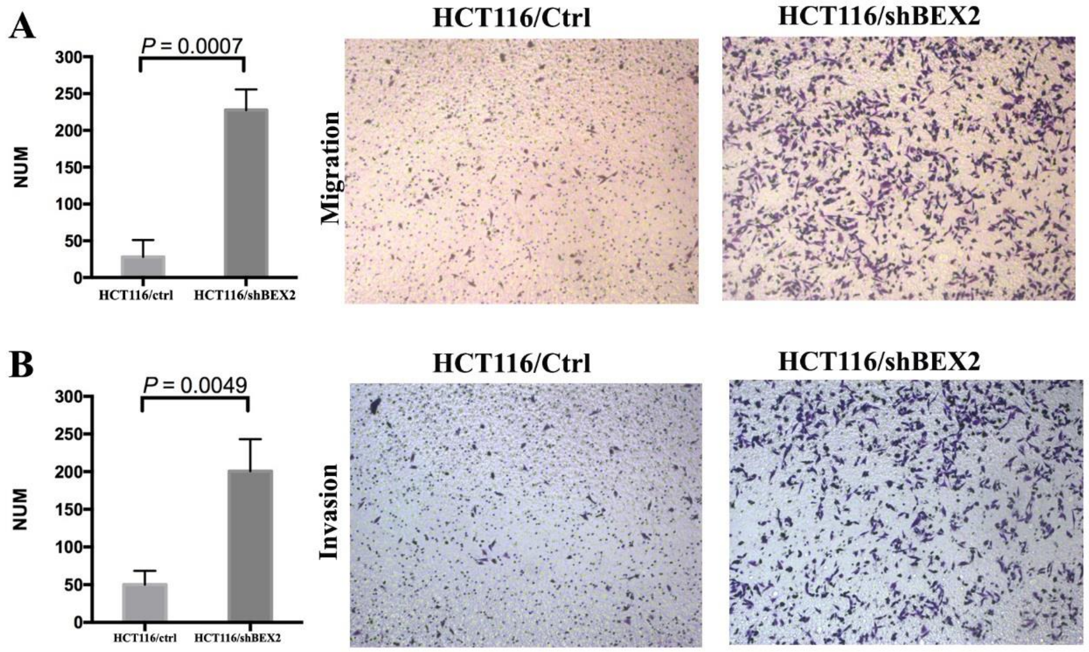

Supplementary Figure 2. BEX2 knockdown enhances migration and invasion in CRC cells. (A) Cell migration assays using Transwell membranes. (left panel, average counts from five random microscopic fields; right panel, representative images of invasion chambers) (magnification, ×100) (B) Cell invasion assays using Matrigel-precoated Transwell membranes (left panel, average counts from five random microscopic fields; right panel, representative images of invasion chambers) and HCT116/shBEX2 cells and HCT116/ctrl cells. (magnification, ×100)

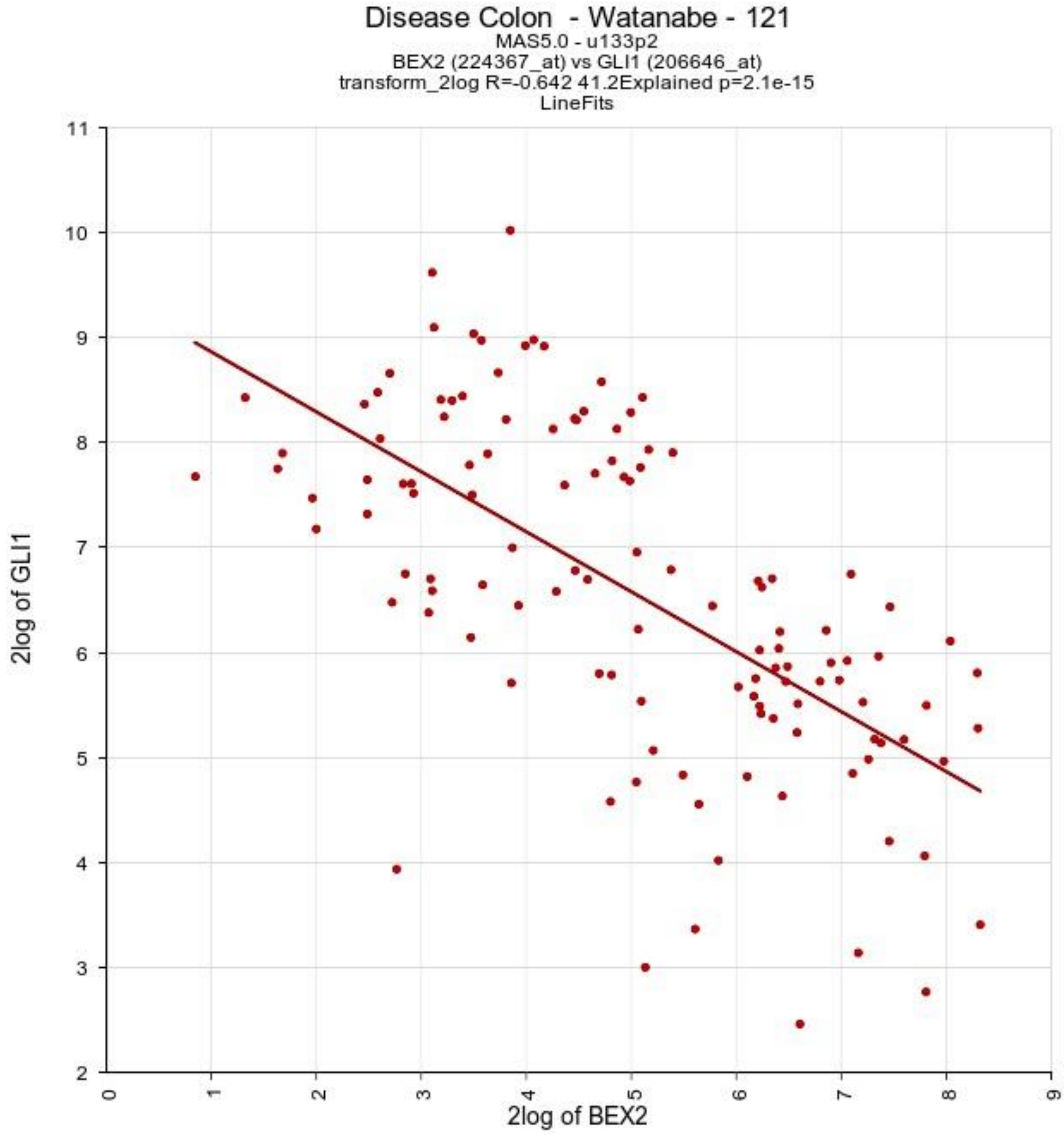

Supplementary Figure 3. BEX2 expression was negatively correlated with GLI1 expression (R value=-0.642, p value=2.1e-15) in GSE3629 data. Figure was obtained from the following website: 'R2: Genomics Analysis and Visualization Platform (http://r2.amc.nl)'.

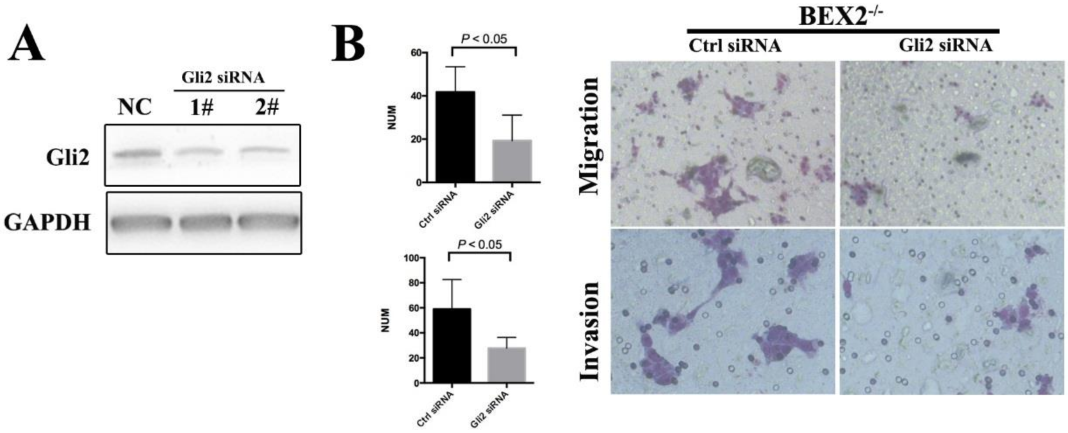

Supplement Figure 4. Gli2 knockdown abrogated the enhanced migration ability in BEX2$^{-/-}$ DLD1 cells

(A) Western blotting for Gli2 after treatment with Gli2 siRNA or normal control (NC) siRNA

(B) Cell migration and invasion assays using Transwell membranes after control siRNA or Gli2 siRNA treatment.

Supplementary Table 1. Primers used for qPCR

Gene	Forward Primer	Reverse Primer
GLI2	CTGCCTCCGAGAAGCAAGAAG	GCATGGAATGGTGGCAAGAG
PTCH1	GAAGAAGGTGCTAATGTCCTGAC	GTCCCAGACTGTAATTTCGCC
PTCH2	GCTTCGTGCTTACTTCCAGGG	CATGCGGAGACCTAATGCCA
SMO	GAAGTGCCCTTGGTTCGGA	GCAGGGTAGCGATTCGAGTT
GLI1	AGCGTGAGCCTGAATCTGTG	CAGCATGTACTGGGCTTTGAA
ZIC2	GCGCAACTCCACAACCAGTA	TGCCGCATATAGCGGAAAAAG
BEX2	TGCCTAGAGGAAACCGTAGG	TCCATCAGCTGTCTCACCTC
GAPDH	CCACTCCTCCACCACCTTTGAC	ACCCTGTTGCTGTAGCCA

Supplementary Table 2. Primary antibodies for Western blot

Gene	Product code	Company	Country

GLI2	18989-1-AP	Proteintech	USA
PTCH1	C53A3	Cell signaling technology (CST)	USA
PTCH2	G1191	CST	USA
SMO	sc-166685	Santa cruz	USA
GLI1	ab49314	Abcam	UK
ZIC2	ab150404	Abcam	UK
BEX2	HPA045384	Sigma	USA
GAPDH	AF11186	Beyotime	China
PCNA	ab29	Abcam	UK

Supplementary Table 3. Quantitative analysis of liver metastases in control and shBEX2 mice

	Number of mice with liver metastases	Number of metastases
SW620/Ctrl	1	4
SW620/shBEX2	4	1
